# Supplementary material for: Phase similarity between similar objects indicates representational merging across retrieval training but not sleep
Source: Imaging Neurosci (Camb). 2026 Jul 31;4:IMAG.a.1321. doi: 10.1162/IMAG.a.1321 (PMC13430988; doi:10.1162/IMAG.a.1321)
Supplement: Supplementary Material [file IMAG.a.1321_supp.pdf]

Supplementary Material 1. Screening questions.

1. What is your gender? (Woman/Man/Non-Binary/None of the above)
2. What is your age in years?
3. Are you a fluent English speaker? (Yes/No)
4. Do you have a history of, or any diagnosed, psychiatric or sleep illnesses (e.g., depression, ADHD, insomnia)? (Yes/No)
5. Have you taken recreational drugs in the last 6 months? (Yes/No)
6. Do you currently take any medication? If so, please enter the name/s. (Yes/No)
7. Do you currently take any medication? If so, please enter the name/s. (Yes/No)
8. Do you have normal/corrected vision? (Yes/No)
9. Do you have normal/corrected hearing? (Yes/No)

Supplementary Material 2. Sample characteristics.

S2 Table A. Questionnaire scores.

Questionnaire	Mean (SD)	Range
Pittsburgh Sleep Quality Index	4.13 (1.11)	1–5
Morningness-Eveningness Questionnaire	50.70 (10.31)	36–73
Flinders Handedness Survey	9.70 (0.79)	7–10

Note. SD = standard deviation.

IAF and custom frequency band descriptives.

IAF: *M*=9.84, *SD*=0.73, range=8.50-11.19.

S2 Table B. Custom frequency ranges.

Frequency band	Lower limit mean (SD)	Upper limit mean (SD)
Theta	4.03 (0.51)	6.20 (0.74)
Alpha	8.13 (0.96)	12.40 (1.48)
Sigma	12.40 (1.48)	16.25 (1.93)
Beta	16.25 (1.93)	24.80 (2.96)

S2 Table C. Sleep intervention characteristics.

Sleep Measure	Mean (SD)	Range
TST (min)	82.44 (26.31)	25.00–116.00
SOL (min)	11.90 (11.11)	1.50–53.00
N1 (%)	16.70 (9.54)	5.00–49.50
N2 (%)	43.90 (14.00)	19.50–71.00
SWS (%)	19.00 (18.31)	0.00–56.00
REM (%)	2.87 (6.30)	0.00–20.50

Note. TST = total sleep time, SOL = sleep onset latency, N1 = stage 1 non-rapid eye movement (NREM) sleep, N2 = stage 2 NREM sleep, SWS = slow-wave sleep, REM = rapid eye movement sleep. No subject experienced sleep onset REM.

Supplementary Material 3. Recognition accuracy model outputs.

S3 Table A. Delayed recognition d prime ~ condition + Immediate recognition d prime + (1|ID)

Effect	χ^2	*df*	*p*
condition	137.24	3	<.001
dprime_ir	129.53	1	<.001

Note. df = degrees of freedom.

S3 Table B. Recognition accuracy change score ~ condition * object_type + (1|ID)

Effect	χ^2	*df*	*p*
condition	56.31	3	<.001
object_type	5.27	2	.072
condition:object_type	35.49	6	<.001

Supplementary Material 4. Object-specific cluster results.

For the first of our EEG analyses, we wanted to identify the times, frequencies, and channels contributing to object-specific representations from learning to immediate recognition. To do this, we first conducted a cluster-based permutation on the phase similarity for the same vs. different object comparison, to capture representations unique to objects. One significant cluster was detected where EEG phase was significantly more similar between same-objects compared to different-objects between learning and immediate recognition (p=.001). This cluster, visualised in the subfigure a, involved phase similarity in the frequencies 2−15 Hz, 20−580 ms post-object onset, across parietal and occipital channels. Next, we conducted another cluster-based permutation for the same-similar object comparison to detect a cluster of representations unique to object, beyond the conceptual likeness they share with their MST similar lures. This revealed one significant cluster (see subfigure c) where phase similarity was greater for same-object comparisons over similar-object comparisons between learning and immediate recognition (p=.036). This cluster encompassed frequencies spanning 2−27 Hz, across the entire epoch (−100−700 ms), with a broad distribution of channels across the entire scalp.

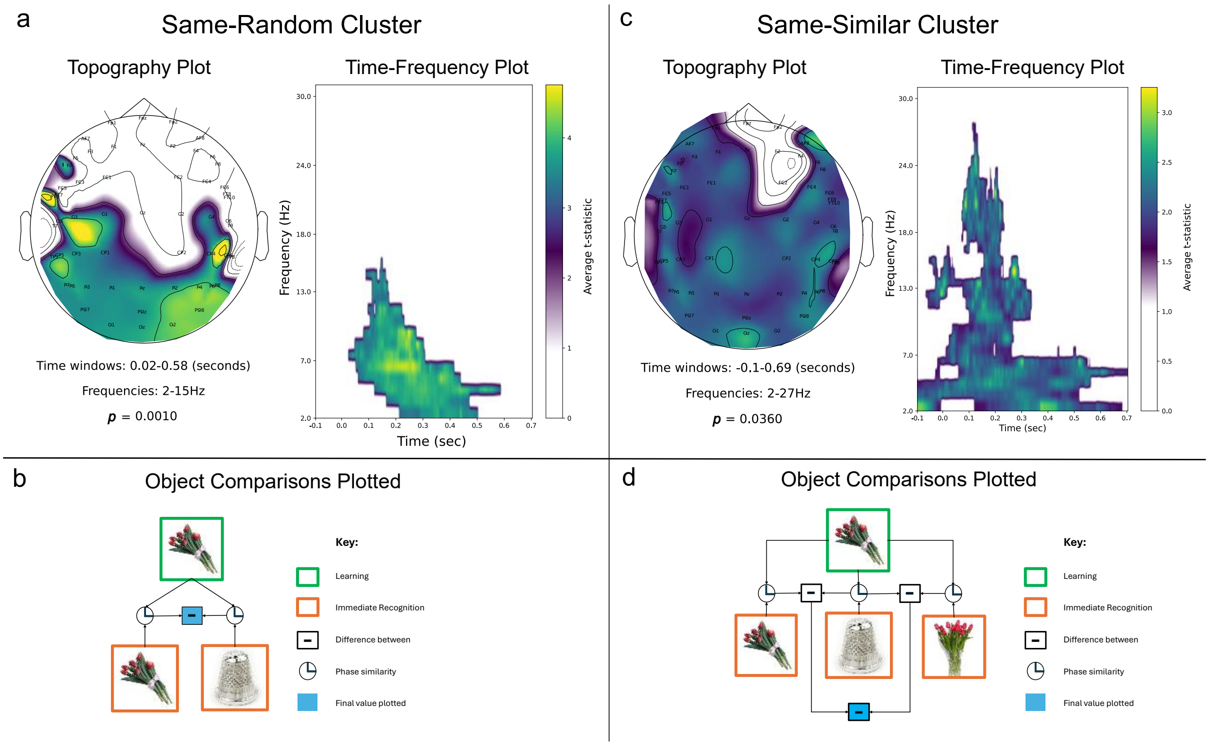

S4 Fig A. Same-different and same-similar object phase similarity clusters. a) This section depicts the cluster of phase similarity scores between learning and immediate recognition comparison of same and different objects. The statistic was computed from a cluster-based permutation (forming clusters across time, frequencies, and channels). The topography plot shows the channels contributing to the cluster, averaged over times and frequencies. The time-frequency plot shows the times and frequencies contributing to the cluster, averaged over channels. b) Illustration of the same-different object comparison performed in the representational similarity analysis plotted in subfigure a. c) Cluster of phase similarity difference between same and similar objects. The statistic was computed from a cluster-based permutation (forming clusters across time, frequencies, and channels). The topography plot shows the channels contributing to the cluster, averaged over times and frequencies. The time-frequency plot shows the times and frequencies contributing to the cluster, averaged over channels. d) Illustration of the same-similar object comparison underlying subfigure c.

Supplementary Material 5. Encoding shift outputs.

S5 Table A. Linear model output – encoding shift.

phase_diff ~ band * time_win + (1|ID) + (1|ch_name)

Effect	χ^2	*df*	*p*
band	92.77	3	<.001
time_win	10.09	1	.001
band:time_win	28.25	3	<.001

Note. df = degrees of freedom.

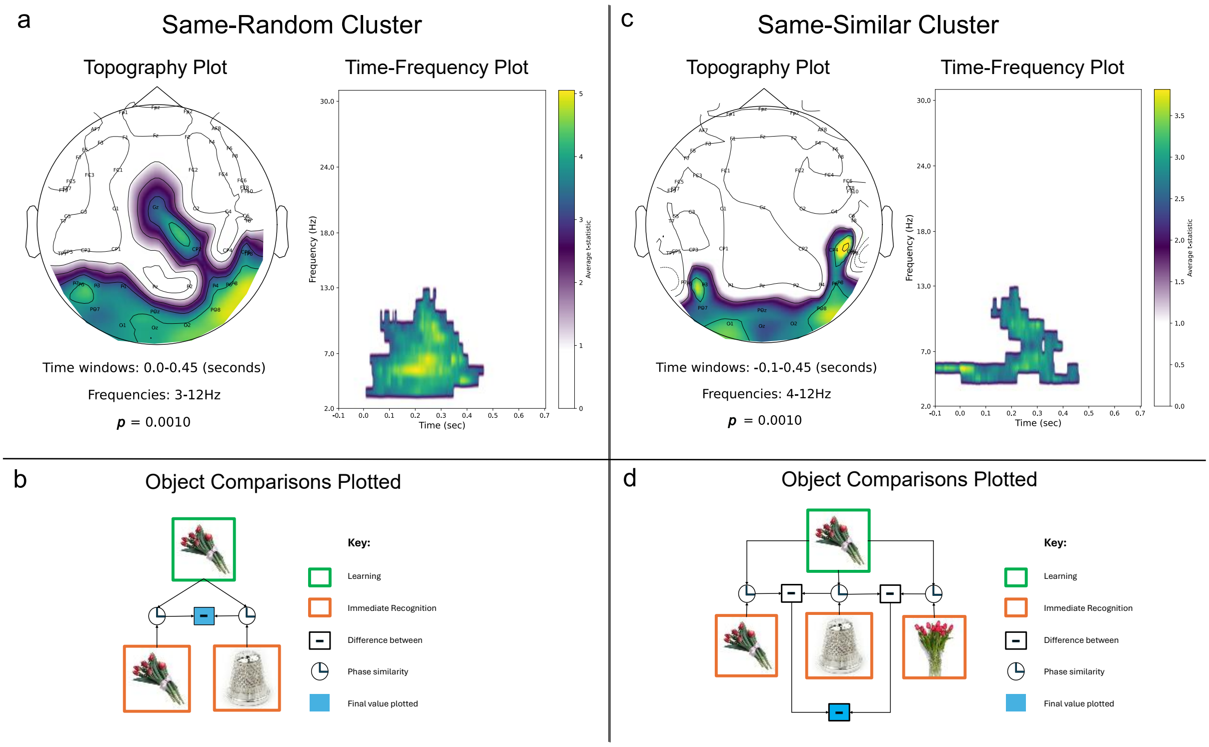

S5 Fig A. Same-different and same-similar object phase similarity clusters, for the average referenced data. a) This section depicts the cluster of phase similarity difference scores determined from the cluster-based permutation across time, frequencies, and topography, for the comparison of same and different objects. The topography plot shows the channels contributing to the cluster, averaged over time and frequencies. The time-frequency plot shows the times and frequencies contributing to the cluster, averaged over channels. b) This key is a simplification of the calculations performed to get the z-scores that are plotted in the cluster graphs, for the same-different object comparison. c) This section depicts the cluster of phase similarity difference scores determined from the cluster-based permutation across time, frequencies, and topography, for the comparison of same and similar objects. The

topography plot shows the channels contributing to the cluster, averaged over time and frequencies. The time-frequency plot shows the times and frequencies contributing to the cluster, averaged over channels. d) This key is a simplification of the object comparisons performed to get the z-scores that are plotted in the cluster graphs, for the same-similar object comparison.

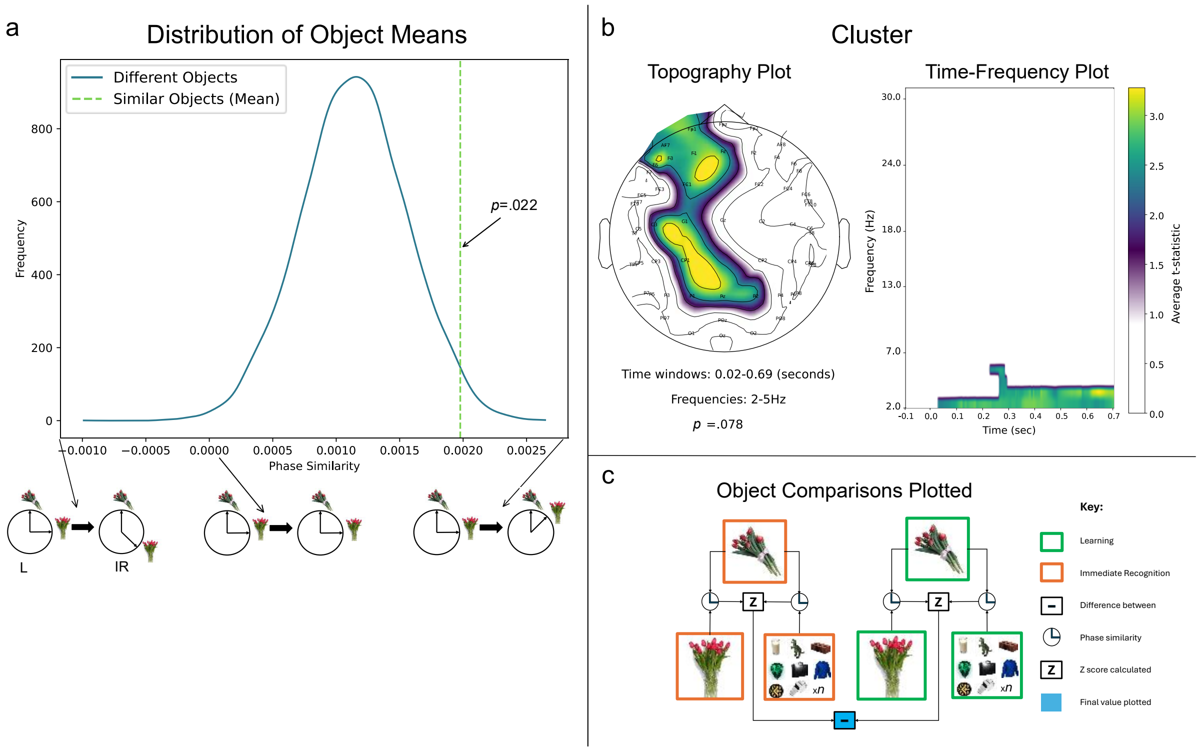

S5 Fig B. Representational merging from learning to immediate recognition, for the average referenced data. L = learning, IR = immediate recognition. a) The ratio highlighted in the distribution of object means refers to the ratio of different-object means that fall above the similar-object mean. The arrows and diagrams along the x-axis of the distribution graph graphically depict the direction of change in phase similarity that occurs from learning to immediate recognition. Negative values indicate decreased phase similarity of similar objects at immediate recognition, positive values indicate increased phase similarity of similar objects at immediate recognition, and values closer to 0 indicate little change from learning to immediate recognition. b) This section depicts the lowest *p*-value cluster of representational change z-scores determined from the cluster-based permutation across time, frequencies, and topography, for the encoding shift. The topography plot shows the channels contributing to the same cluster, averaged over time and frequencies. The time-frequency plot shows the times and frequencies contributing to the cluster, averaged over channels. c) This key is a

simplification of the object comparisons performed to get the z-scores that are plotted in the cluster plots.

S5 Table B. The sources contributing to representational merging in the encoding shift.

Region	Z-score
Cuneal Cortex*	3.610286
Occipital Pole	3.433389
Occipital Pole	3.368327
Cuneal Cortex	3.287755
Cuneal Cortex	3.284609
Precuneous Cortex*	3.283758
Cuneal Cortex	3.23431
Precuneous Cortex	3.175432
Lateral Occipital Cortex, superior division*	3.088707
Precuneous Cortex	3.048152
Occipital Pole	3.015727
Cuneal Cortex	2.990822
Lateral Occipital Cortex, superior division*	2.952351
Precuneous Cortex	2.937046
Lateral Occipital Cortex, superior division	2.90953
Lingual Gyrus	2.902009
Temporal Occipital Fusiform Cortex*	2.883048
Lateral Occipital Cortex, superior division	2.868235
Occipital Pole	2.857403
Lateral Occipital Cortex, superior division*	2.844147
Precuneous Cortex	2.80497
Intracalcarine Cortex	2.793041
Occipital Pole	2.777427
Cuneal Cortex	2.759317
Temporal Occipital Fusiform Cortex*	2.755286
Precuneous Cortex	2.738626
Lateral Occipital Cortex, superior division	2.706178

Temporal Occipital Fusiform Cortex*	2.696939
Occipital Fusiform Gyrus*	2.681206
Intracalcarine Cortex*	2.674155
Lateral Occipital Cortex, superior division	2.652494
Supracalcarine Cortex	2.645354
Angular Gyrus*	2.63934
Precentral Gyrus	2.622412
Lateral Occipital Cortex, superior division	2.609404
Occipital Pole*	2.599115
Occipital Fusiform Gyrus*	2.595404
Occipital Fusiform Gyrus*	2.595057
Lateral Occipital Cortex, superior division	2.592317
Occipital Pole*	2.582396
Lateral Occipital Cortex, superior division	2.579731
Occipital Fusiform Gyrus*	2.579388
Precuneous Cortex	2.578065
Lateral Occipital Cortex, inferior division*	2.574267
Cingulate Gyrus, posterior division*	2.573091
Cingulate Gyrus, posterior division*	2.569154
Superior Frontal Gyrus	2.566944
Precuneous Cortex	2.559386
Lateral Occipital Cortex, superior division	2.55751
Precentral Gyrus	2.55312
Precuneous Cortex	2.551836
Occipital Fusiform Gyrus*	2.549871
Precuneous Cortex	2.545103
Occipital Pole	2.527269
Precentral Gyrus	2.523482
Precuneous Cortex	2.520608
Occipital Fusiform Gyrus*	2.518736
Precuneous Cortex	2.515015
Intracalcarine Cortex	2.48857

Intracalcarine Cortex	2.487951
Precuneous Cortex	2.480163
Occipital Pole	2.479822
Temporal Occipital Fusiform Cortex*	2.472744
Lingual Gyrus	2.47223
Temporal Occipital Fusiform Cortex*	2.464579
Intracalcarine Cortex	2.460055
Superior Parietal Lobule*	2.443011
Precentral Gyrus	2.439102
Temporal Occipital Fusiform Cortex	2.434801
Occipital Fusiform Gyrus*	2.428142
Frontal Orbital Cortex	2.427346
Occipital Fusiform Gyrus*	2.42669
Precuneous Cortex*	2.424383
Parietal Opercular Cortex*	2.415987
Precuneous Cortex*	2.40812
Occipital Fusiform Gyrus	2.406016
Occipital Pole	2.405976
Postcentral Gyrus	2.403178
Cuneal Cortex*	2.402296
Precuneous Cortex*	2.397974
Precuneous Cortex*	2.394977
Parietal Opercular Cortex*	2.389892
Lingual Gyrus*	2.387962
Occipital Pole	2.385467
Precuneous Cortex*	2.385415
Intracalcarine Cortex*	2.381131
Precuneous Cortex*	2.373169
Occipital Fusiform Gyrus*	2.37148
Lingual Gyrus	2.370085
Lingual Gyrus*	2.348929
Frontal Pole	2.348588

Occipital Fusiform Gyrus	2.343224
Occipital Fusiform Gyrus*	2.338669
Lateral Occipital Cortex, superior division	2.334747
Precuneous Cortex	2.327487
Temporal Occipital Fusiform Cortex*	2.324217
Intracalcarine Cortex	2.321942
Temporal Occipital Fusiform Cortex*	2.315797
Precuneous Cortex	2.309127
Parietal Opercular Cortex*	2.308489
Intracalcarine Cortex	2.306691
Lateral Occipital Cortex, inferior division	2.30664
Temporal Occipital Fusiform Cortex	2.302886
Lateral Occipital Cortex, superior division*	2.301408
Temporal Occipital Fusiform Cortex*	2.301285
Temporal Occipital Fusiform Cortex*	2.299544
Precuneous Cortex*	2.297307
Postcentral Gyrus	2.297101
Precuneous Cortex*	2.29261
Occipital Pole*	2.289288
Occipital Fusiform Gyrus*	2.288041
Occipital Pole*	2.283177
Occipital Pole*	2.282057
Lingual Gyrus	2.280789
Precuneous Cortex	2.280109
Angular Gyrus*	2.274413
Occipital Pole*	2.273088
Lingual Gyrus	2.272555
Precentral Gyrus	2.272482
Lingual Gyrus*	2.269038
Lateral Occipital Cortex, superior division	2.267463
Lateral Occipital Cortex, inferior division	2.264558
Supramarginal Gyrus, posterior division	2.264343

Temporal Occipital Fusiform Cortex*	2.264127
Superior Frontal Gyrus	2.260452
Precuneous Cortex	2.259709
Lingual Gyrus*	2.257329
Precuneous Cortex	2.256884
Occipital Pole	2.254788
Temporal Occipital Fusiform Cortex	2.253465
Occipital Pole	2.249175
Temporal Occipital Fusiform Cortex*	2.248874
Cingulate Gyrus, posterior division*	2.245007
Cingulate Gyrus, posterior division*	2.244764
Temporal Occipital Fusiform Cortex*	2.237692
Lingual Gyrus*	2.232709
Temporal Pole	2.230007
Occipital Pole	2.22491
Postcentral Gyrus	2.21814
Lingual Gyrus*	2.217523
Precentral Gyrus	2.217469
Intracalcarine Cortex*	2.210054
Superior Frontal Gyrus	2.208882
Superior Parietal Lobule	2.207019
Occipital Pole	2.206722
Parietal Opercular Cortex*	2.206428
Lingual Gyrus*	2.197507
Postcentral Gyrus	2.195352
Frontal Orbital Cortex*	2.194345
Central Opercular Cortex*	2.193932
Intracalcarine Cortex*	2.19063
Precuneous Cortex*	2.187564
Occipital Fusiform Gyrus*	2.182645
Occipital Fusiform Gyrus*	2.181466
Lateral Occipital Cortex, superior division*	2.181196

Superior Parietal Lobule	2.180386
Superior Temporal Gyrus, anterior division	2.176113
Precentral Gyrus	2.175257
Temporal Occipital Fusiform Cortex	2.173441
Intracalcarine Cortex	2.17328
Occipital Fusiform Gyrus	2.170551
Occipital Pole*	2.169599
Temporal Occipital Fusiform Cortex*	2.166143
Occipital Pole	2.162568
Superior Temporal Gyrus, anterior division	2.160326
Cuneal Cortex*	2.159313
Occipital Pole	2.152942
Angular Gyrus	2.150757
Lingual Gyrus	2.148802
Lingual Gyrus*	2.148143
Precentral Gyrus	2.147429
Lingual Gyrus*	2.147265
Angular Gyrus*	2.146048
Lateral Occipital Cortex, inferior division	2.142894
Angular Gyrus	2.138664
Temporal Occipital Fusiform Cortex*	2.135801
Postcentral Gyrus	2.133471
Frontal Orbital Cortex	2.130683
Parietal Opercular Cortex	2.129339
Intracalcarine Cortex	2.120218
Lateral Occipital Cortex, superior division	2.119805
Cuneal Cortex	2.11571
Precuneous Cortex*	2.108865
Superior Parietal Lobule*	2.106325
Lingual Gyrus*	2.104742
Occipital Pole	2.103745
Precentral Gyrus	2.097006

Lateral Occipital Cortex, inferior division*	2.096444
Precuneous Cortex	2.095755
Postcentral Gyrus	2.093352
Lingual Gyrus*	2.092496
Lingual Gyrus	2.091939
Lingual Gyrus*	2.090645
Occipital Fusiform Gyrus*	2.089395
Angular Gyrus*	2.089243
Lateral Occipital Cortex, inferior division	2.089189
Planum Temporale*	2.08665
Superior Parietal Lobule	2.080675
Lingual Gyrus	2.079484
Temporal Occipital Fusiform Cortex*	2.077324
Precentral Gyrus	2.072901
Lingual Gyrus*	2.072339
Lingual Gyrus*	2.066172
Lateral Occipital Cortex, inferior division	2.065183
Temporal Occipital Fusiform Cortex	2.064712
Lateral Occipital Cortex, inferior division	2.063258
Central Opercular Cortex	2.062391
Lingual Gyrus	2.060863
Superior Frontal Gyrus	2.060813
Lingual Gyrus	2.059546
Occipital Pole*	2.058876
Precuneous Cortex	2.057173
Temporal Occipital Fusiform Cortex*	2.056761
Lingual Gyrus	2.055115
Occipital Fusiform Gyrus	2.054865
Temporal Pole	2.050422
Parietal Opercular Cortex	2.049772
Lingual Gyrus*	2.047438
Lateral Occipital Cortex, superior division*	2.044793

Lateral Occipital Cortex, superior division	2.044502
Temporal Occipital Fusiform Cortex	2.043955
Planum Polare	2.038782
Occipital Pole	2.032952
Temporal Occipital Fusiform Cortex*	2.032297
Insular Cortex	2.030444
Occipital Fusiform Gyrus	2.02996
Occipital Fusiform Gyrus	2.028746
Planum Temporale*	2.027966
Occipital Pole*	2.026629
Occipital Fusiform Gyrus*	2.026454
Lateral Occipital Cortex, inferior division	2.026186
Temporal Occipital Fusiform Cortex*	2.025631
Superior Parietal Lobule*	2.024665
Occipital Fusiform Gyrus*	2.023766
Lateral Occipital Cortex, superior division*	2.021735
Lateral Occipital Cortex, superior division	2.020949
Occipital Pole*	2.019448
Cuneal Cortex	2.019361
Planum Polare	2.018153
Supramarginal Gyrus, anterior division	2.016531
Temporal Occipital Fusiform Cortex*	2.016127
Cingulate Gyrus, posterior division	2.015509
Lateral Occipital Cortex, inferior division	2.015105
Lateral Occipital Cortex, inferior division*	2.013263
Temporal Pole	2.012554
Occipital Pole*	2.010403
Precuneous Cortex*	2.007425
Lateral Occipital Cortex, superior division	2.006966
Intracalcarine Cortex	2.006259
Angular Gyrus	2.004725
Precentral Gyrus	2.003792

Lateral Occipital Cortex, inferior division	2.003447
Lingual Gyrus*	2.001599
Precuneous Cortex	2.000541
Temporal Occipital Fusiform Cortex*	1.99621
Postcentral Gyrus	1.995835
Lateral Occipital Cortex, inferior division*	1.994476
Occipital Fusiform Gyrus*	1.990068
Cingulate Gyrus, posterior division*	1.988349
Superior Temporal Gyrus, anterior division	1.985597
Lateral Occipital Cortex, inferior division*	1.984651
Temporal Occipital Fusiform Cortex*	1.9838
Occipital Fusiform Gyrus*	1.983216
Occipital Pole*	1.981961
Occipital Fusiform Gyrus	1.979451
Temporal Occipital Fusiform Cortex*	1.977084
Precentral Gyrus	1.974328
Lateral Occipital Cortex, superior division	1.973814
Occipital Fusiform Gyrus*	1.973303
Superior Parietal Lobule	1.971548
Lateral Occipital Cortex, superior division	1.970603
Postcentral Gyrus*	1.968411
Lateral Occipital Cortex, superior division	1.968166
Lateral Occipital Cortex, inferior division	1.963858
Lingual Gyrus*	1.963407

Note. * = sources that were assigned the nearest non-background label. The labels were taken from the Harvard-Oxford dictionary, based on the MNI coordinates of the source. The representational change z-scores of each source are all above the 97.5th percentile and are averaged over times and frequencies in the encoding cluster.

S6 File. Intervention shift outputs.

S6 Table A. Linear model output – intervention shift.

phase_diff ~ band * time_win + (1|ID) + (1|ch_name)

Effect	*F*	*df*	*p*
band	116.38	3	<.001
time_win	4.06	1	.044
band:time_win	34.06	3	<.001

Note. df = degrees of freedom.

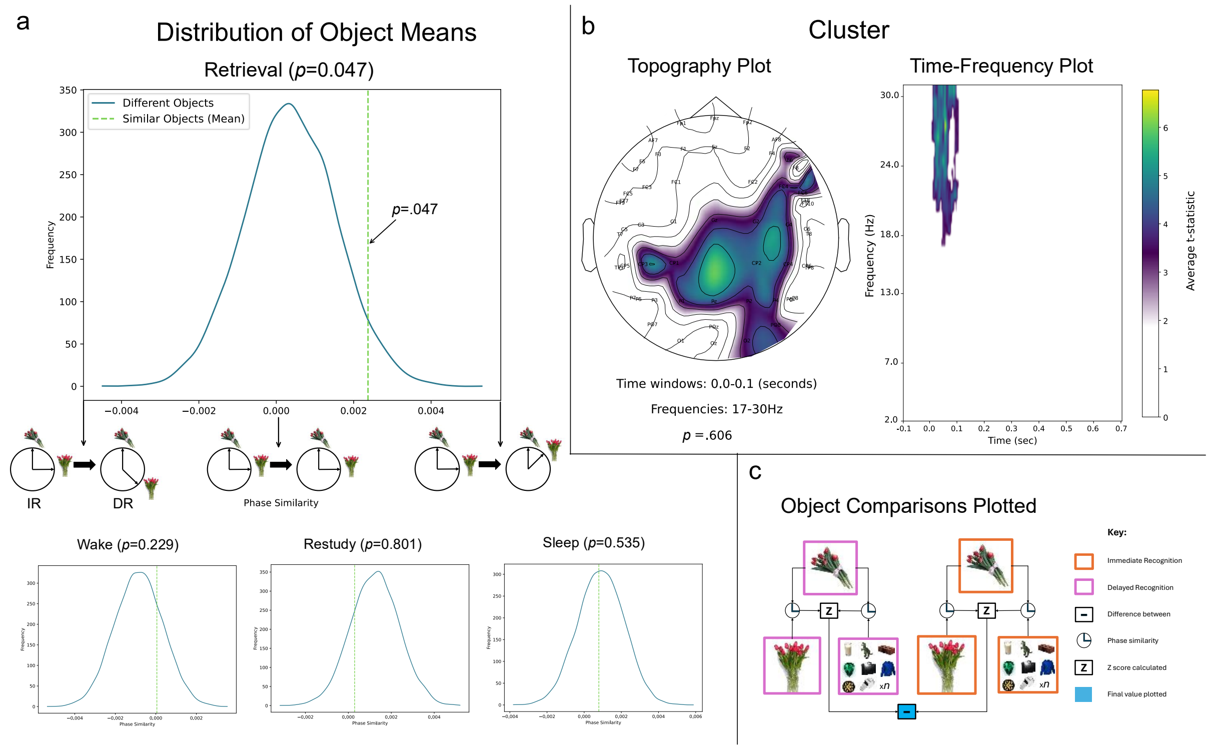

S6 Fig A. Representational merging from immediate to delayed recognition, for the average referenced data. IR = immediate recognition, DR = delayed recognition. a) The ratio highlighted in the distribution of object means refers to the ratio of different-object means that fall above the similar-object mean. The arrows and diagrams along the x-axis of the distribution graph graphically depict the direction of change in phase similarity that occurs from immediate recognition to delayed recognition. Negative values indicate decreased phase similarity of similar objects at delayed recognition, positive values indicate increased phase similarity of similar objects at delayed recognition, and values closer to 0 indicate little change from immediate to delayed recognition. b) This section depicts the lowest *p*-values cluster of representational change z-scores determined from the cluster-based

permutation across time, frequencies, and topography, for the intervention shift. The topography plot shows the channels contributing to this cluster, averaged over time and frequencies. The time-frequency plot shows the times and frequencies contributing to the cluster, averaged over channels. c) This key is a simplification of the object comparisons performed to get the z-scores that are plotted in the cluster plots.

S6 Table B. The sources contributing to representational merging in the retrieval training's intervention shift.

Region	Z-score
Lateral Occipital Cortex, inferior division	2.445826
Parietal Opercular Cortex*	2.427392
Parahippocampal Gyrus, posterior division*	2.331251
Lingual Gyrus*	2.288233
Supramarginal Gyrus, anterior division	2.287396
Inferior Temporal Gyrus, posterior division	2.285315
Parietal Opercular Cortex*	2.275704
Inferior Temporal Gyrus, temporooccipital part*	2.273214
Inferior Temporal Gyrus, posterior division	2.267414
Parietal Opercular Cortex	2.256483
Lingual Gyrus	2.213528
Parahippocampal Gyrus, posterior division*	2.211534
Lateral Occipital Cortex, inferior division	2.187111
Lingual Gyrus*	2.158867
Parietal Opercular Cortex	2.156303
Lingual Gyrus	2.123423
Planum Temporale	2.081967
Temporal Fusiform Cortex, posterior division	2.046327
Intracalcarine Cortex	2.045143
Temporal Fusiform Cortex, posterior division	2.036757
Inferior Temporal Gyrus, temporooccipital part*	2.031056
Planum Temporale*	2.013499
Frontal Pole	2.009094

Postcentral Gyrus	2.005623
Superior Temporal Gyrus, posterior division	2.005462
Superior Parietal Lobule*	1.994609
Lingual Gyrus	1.992909
Inferior Temporal Gyrus, temporooccipital part*	1.987299
Occipital Fusiform Gyrus*	1.981476
Postcentral Gyrus	1.981282
Postcentral Gyrus*	1.980683
Occipital Fusiform Gyrus	1.978575
Parietal Opercular Cortex*	1.977752
Lingual Gyrus*	1.975243
Supramarginal Gyrus, anterior division*	1.972651
Inferior Temporal Gyrus, temporooccipital part*	1.971383
Angular Gyrus*	1.961389
Supramarginal Gyrus, anterior division*	1.957754
Parahippocampal Gyrus, posterior division*	1.946544
Lingual Gyrus	1.944044
Lingual Gyrus*	1.930077
Occipital Fusiform Gyrus*	1.929982
Heschl's Gyrus (includes H1 and H2)*	1.929654
Frontal Pole	1.929601
Occipital Pole	1.928322
Superior Parietal Lobule	1.924446
Supramarginal Gyrus, posterior division	1.918625
Parahippocampal Gyrus, posterior division*	1.918277
Lateral Occipital Cortex, inferior division	1.917294
Central Opercular Cortex*	1.916706
Temporal Fusiform Cortex, posterior division*	1.909189
Inferior Temporal Gyrus, posterior division*	1.902406
Parietal Opercular Cortex	1.888452
Temporal Occipital Fusiform Cortex*	1.880122
Intracalcarine Cortex	1.877455

Occipital Fusiform Gyrus*	1.875197
Lingual Gyrus*	1.871478
Parahippocampal Gyrus, posterior division*	1.870345
Temporal Occipital Fusiform Cortex*	1.867681
Lingual Gyrus*	1.865232
Occipital Pole*	1.859396
Frontal Pole	1.852865
Lingual Gyrus	1.851399
Lingual Gyrus*	1.839977
Planum Polare*	1.838727
Lingual Gyrus	1.834542
Temporal Occipital Fusiform Cortex	1.833787
Occipital Fusiform Gyrus*	1.833051
Precentral Gyrus	1.829572
Inferior Temporal Gyrus, posterior division*	1.828496
Lingual Gyrus*	1.826681
Inferior Temporal Gyrus, posterior division	1.820967
Occipital Fusiform Gyrus*	1.81754
Lingual Gyrus	1.817463
Occipital Fusiform Gyrus*	1.810418
Intracalcarine Cortex	1.808598
Occipital Fusiform Gyrus	1.798365
Occipital Fusiform Gyrus	1.797566
Lateral Occipital Cortex, superior division*	1.79659
Occipital Fusiform Gyrus*	1.79543
Parahippocampal Gyrus, posterior division	1.792443
Inferior Temporal Gyrus, temporooccipital part	1.789034
Occipital Fusiform Gyrus*	1.788195
Occipital Pole*	1.780354
Parahippocampal Gyrus, posterior division*	1.779156
Occipital Fusiform Gyrus*	1.776932
Lateral Occipital Cortex, inferior division*	1.772577

Parahippocampal Gyrus, posterior division	1.770781
Lateral Occipital Cortex, inferior division	1.770607
Occipital Pole	1.767734
Lingual Gyrus	1.766744
Occipital Fusiform Gyrus*	1.765145
Occipital Fusiform Gyrus*	1.762489
Lateral Occipital Cortex, inferior division*	1.761022
Heschl's Gyrus (includes H1 and H2)	1.760993
Temporal Occipital Fusiform Cortex	1.760453
Occipital Fusiform Gyrus*	1.756024
Temporal Fusiform Cortex, posterior division*	1.752779
Occipital Pole	1.750047
Temporal Occipital Fusiform Cortex*	1.744751
Planum Temporale*	1.741924
Lateral Occipital Cortex, inferior division	1.740078
Supramarginal Gyrus, anterior division*	1.73724
Inferior Temporal Gyrus, temporooccipital part*	1.731544
Occipital Fusiform Gyrus*	1.727756
Lingual Gyrus	1.725413
Postcentral Gyrus	1.722821
Lingual Gyrus	1.722529
Superior Parietal Lobule	1.721437
Occipital Fusiform Gyrus*	1.718859
Occipital Pole*	1.713911
Lingual Gyrus*	1.70741
Middle Temporal Gyrus, temporooccipital part	1.702911
Temporal Occipital Fusiform Cortex*	1.7015
Temporal Occipital Fusiform Cortex*	1.698469
Lingual Gyrus*	1.695383
Occipital Pole*	1.695002
Occipital Pole	1.692981
Occipital Pole*	1.691381

Planum Temporale	1.688459
Supramarginal Gyrus, anterior division	1.687475
Middle Temporal Gyrus, temporooccipital part	1.687317
Lateral Occipital Cortex, inferior division*	1.686277
Lingual Gyrus*	1.685822
Occipital Fusiform Gyrus	1.6844
Lingual Gyrus*	1.682082
Parahippocampal Gyrus, posterior division*	1.680892
Inferior Temporal Gyrus, temporooccipital part*	1.6796
Lingual Gyrus*	1.679082
Temporal Occipital Fusiform Cortex*	1.676162
Intracalcarine Cortex*	1.675666
Occipital Pole	1.672823
Supramarginal Gyrus, anterior division*	1.672066
Lateral Occipital Cortex, inferior division	1.671601
Lingual Gyrus*	1.669688
Lingual Gyrus*	1.667106
Temporal Occipital Fusiform Cortex*	1.666532
Temporal Fusiform Cortex, posterior division*	1.664836
Lingual Gyrus*	1.662175
Parahippocampal Gyrus, anterior division*	1.661382
Parahippocampal Gyrus, posterior division*	1.66135
Occipital Pole	1.661072
Lingual Gyrus*	1.659795
Parahippocampal Gyrus, posterior division*	1.65873
Occipital Pole*	1.656698
Occipital Pole	1.656061
Postcentral Gyrus	1.652582
Intracalcarine Cortex*	1.651714
Temporal Occipital Fusiform Cortex*	1.650413
Occipital Fusiform Gyrus*	1.648898
Cingulate Gyrus, posterior division*	1.647916

Occipital Fusiform Gyrus*	1.646271

Note. * = sources that were assigned the nearest non-background label. The labels were taken from the Harvard-Oxford dictionary, based on the MNI coordinates of the source. The representational change z-scores of each source are all above the 95th percentile and are averaged 400–700 ms and 7–13 Hz.

S7 File. Session-level behaviour modelling.

We aimed to test if the change in propensity for subjects to endorse similar lures from pre- to post-intervention, differed based on the intervention condition. To address this, we conducted a linear mixed-effects model predicting subjects' similar-different change scores (i.e., the change in similar- over different-lure accuracy, from immediate to delayed recognition), using the intervention condition as a fixed effect. This model revealed a significant effect of condition, $\chi^2(3)=18.47$, $p<.001$, which is displayed in Fig A. This figure illustrates that the propensity to endorse similar lures more than different lures generally increased across the retrieval training and wake interventions, but decreased across the sleep and restudy periods. Treatment contrast coding revealed that retrieval training and sleep conditions did not differ significantly.

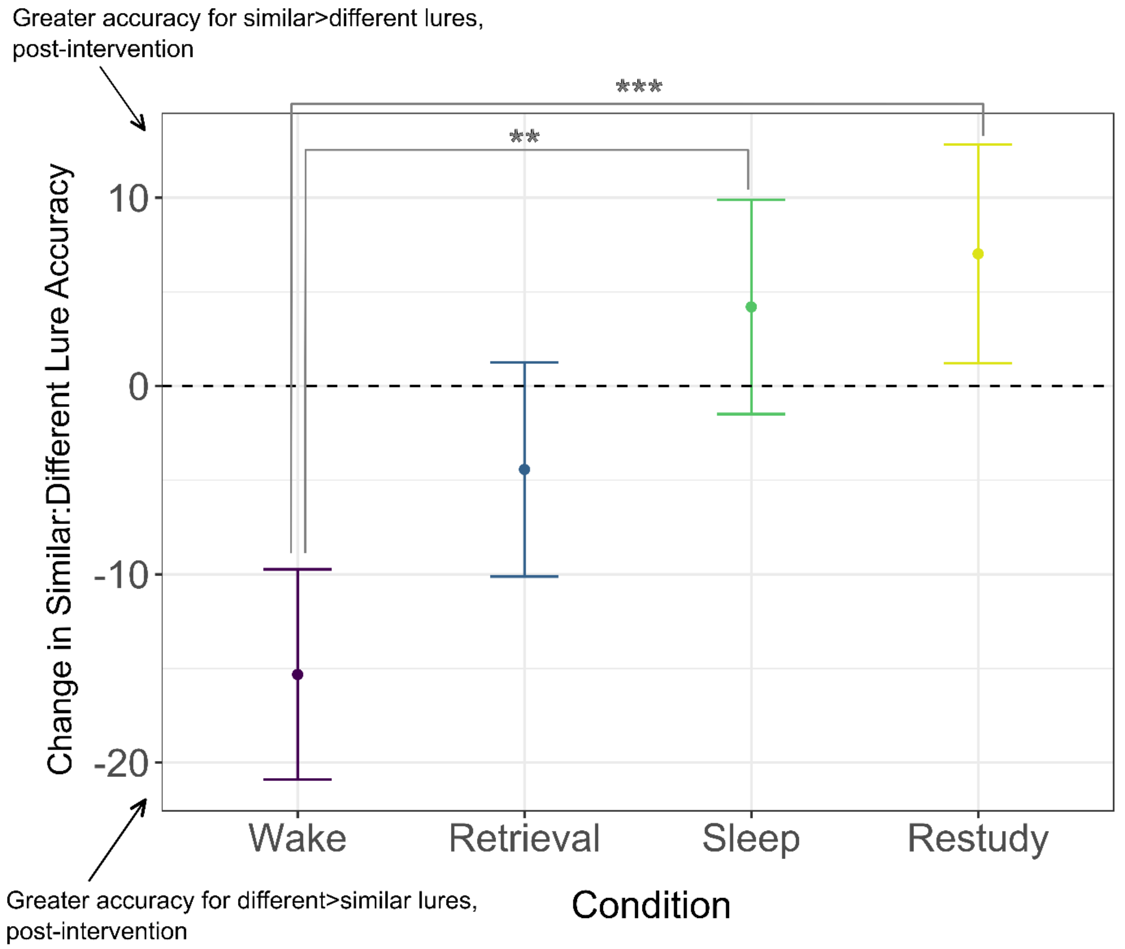

S7 Fig A. Condition differences for the change in similar-lure over different-lure accuracy. The y-axis represents the difference between similar-lure accuracy and different-

lure accuracy, from immediate to delayed recognition. Positive numbers indicate that there was a greater accuracy for similar- over different-object lures post-intervention, compared to pre-intervention. Negative numbers indicate that there was a greater accuracy for different over similar lures post-intervention, compared to pre-intervention. The zero point is marked with a dotted line, indicating little to no change in the difference between similar- over different lure accuracy across the intervention. The error bars represent the 83% confidence intervals. Significant contrasts are marked as: *denotes $p<.05$, **$p<.01$, and ***$p<.001$.

Additionally, we used the representational change z-scores (averaged over the 400–700ms alpha-band) to predict subjects' similar-different change scores (i.e., the change in session-level endorsement of similar lures over different lures, across the intervention period), for the retrieval training intervention. The purpose of this was to have an analogous behaviour measure of representational change to our neural measure. The linear model regression predicting similar-different change scores across retrieval training from the representational change z-scores, did not yield statistical significance $F(1)=0.004$, $p=.950$.
